# SMAD4 enhances the cytotoxic efficacy of human NK cells against colorectal cancer cells via the m^6^A reader YTHDF2

**DOI:** 10.3389/fimmu.2024.1440308

**Published:** 2024-10-07

**Authors:** Xinxin Li, Yilin Wang, Lei Cai, Siyong Huang

**Affiliations:** ^1^ Xi'an Key Laboratory of Stem Cell and Regenerative Medicine, Institute of Medical Research, Northwestern Polytechnical University, Xi’an, Shaanxi, China; ^2^ Research & Development Institute of Northwestern Polytechnical University in Shenzhen, Shenzhen, Guangdong, China; ^3^ Department of Psychiatry, Zigong Mental Health Center, the Zigong Affiliated Hospital of Southwest Medical University, Zigong, Sichuan, China; ^4^ Division of Digestive Surgery, Hospital of Digestive Diseases, Xi’an International Medical Center, Xi’an, Shaanxi, China; ^5^ Department of Hematology, Xi’an International Medical Center, Xi’an, Shaanxi, China

**Keywords:** SMAD4, human NK cells, colorectal cancer cell, cytotoxicity, YTHDF2

## Abstract

**Background:**

Colorectal cancer (CRC) ranks as the third most prevalent malignant neoplasm in terms of both morbidity and mortality. Within the tumor microenvironment (TME) of CRC, the diminished presence and diminished cytotoxic function of natural killer (NK) cells serve as important factors driving the advancement of CRC; however, the precise regulatory mechanisms governing this phenomenon remain incompletely understood. Consequently, the identification of novel, potential anti-CRC targets associated with NK cells emerges as a pressing and paramount concern warranting immediate attention.

**Methods:**

We examined the regulatory mechanism of SMAD4-mediated NK cell cytotoxicity on CRC by utilizing various experimental techniques, such as qRT-PCR, flow cytometry.

**Results:**

Our findings revealed that the expression of SMAD4 is decreased in NK cells within the TME of human CRC. Furthermore, we observed that enforced upregulation of SMAD4 resulted in enhanced cytotoxicity of NK cells towards CRC cells. Furthermore, our research has revealed that YTHDF2 functions as a downstream effector of SMAD4, playing a crucial role in the control of transcription and translation of m^6^A-modified RNA. Moreover, our investigation demonstrated that increased expression of SMAD4 promoted the activating receptor NKG2D by elevating levels of YTHDF2. Ultimately, the SMAD4-YTHDF2 regulatory axis significantly enhanced the cytotoxicity of NK cells against human CRC cells.

**Conclusion:**

Our study unveils a novel mechanism through which SMAD4 modulates the cytotoxicity of NK cells towards CRC cells, suggesting that SMAD4 may hold promise as a potential therapeutic target for NK cell therapy in CRC.

## 1 Introduction

Colorectal cancer (CRC) ranks as the third most frequently diagnosed malignancy, characterized by significant morbidity and mortality rates (1). Over recent decades, advancements in the utilization of small molecular chemotherapy agents (e.g., Cetuximab) and immune checkpoint inhibitors (e.g., PD-1/PDL-1) have shown improved therapeutic outcomes in a subset of CRC patients (2, 3). Nevertheless, the therapeutic efficacy for patients with relapsed or drug-resistant CRC remains suboptimal. Consequently, the identification of novel targets for CRC represents a pressing and pivotal concern that warrants immediate attention.

Within the tumor microenvironment (TME) of CRC, immune cells exhibit a dual regulatory function by either impeding or promoting tumor progression (4). Notably, activated natural killer (NK) cells are a crucial component of the immune response, directly targeting and eliminating CRC (5, 6). The utilization of NK cell therapy, such as chimeric antigen receptor (CAR)-NK cells, has emerged as a promising strategy in the realm of CRC immunotherapy in recent years (7–9). NK cells can be activated by tumor cells, cytokines [e.g., interleukin (IL)-2, IL-12], natural products (e.g., chitosan) to induce direct lysis of tumor cells through the secretion of toxic molecules [e.g., granzyme B (GZMB) and perforin (PRF1)] (10–12). Additionally, NK cells can also secrete lymphokines [e.g., interferon-gamma (IFN-γ)] to modulate immune responses, indirectly impeding tumor cell proliferation and metastasis (13). Previous research has demonstrated that the cytotoxic function of NK cells can be triggered by the overexpression of ligands [e.g., poliovirus receptor (CD155), nectin-2 (CD112)] on the surface of human CRC cells (14). Nevertheless, the specific molecular mechanisms governing the regulation of NK cell cytotoxicity on CRC cells remain largely unexplored.

As investigations into tumor immunotherapy progress, the influence of mothers against decapentaplegic homolog 4 (SMAD4) on immune cells is increasingly coming to light. SMAD4, a member of the SMAD family, serves as a mediator for membrane receptors involved in regulating SMAD signaling (15). SMAD4 has the capability to form heterologous complexes with other members of the activated SMAD family, facilitating their translocation to the nucleus where they collaboratively regulate the expression of target genes alongside other transcription factors (16). Huss et al. have demonstrated that SMAD4 promotes the secretion of IL-10 from T helper type 1(Th1) cells, leading to a reduction in encephalitogenic markers (e.g., T-bet) on IL-10R-expressing Th1 effector cells and inhibiting the recruitment of inflammatory T cells (17). Furthermore, Hu et al. have shown that SMAD4 plays a crucial role in the differentiation of effector and circulating memory CD8^+^ T cells (18). Two studies conducted on mouse NK cells have demonstrated that Smad4 play a role in promoting the maturation of mouse NK cells and inhibiting the metastasis of melanoma, regardless of TGF-β signaling (16). Conversely, the absence of Smad4 in NK cells has been shown to enhance the metastasis of melanoma cells and viral infection (19). These findings suggest that mouse Smad4 not only facilitates the development of NK cells, but also strengthens their anti-tumor capabilities. Also in tumor TME, the role of SMAD4 in tumors and tumor immunity is complicated, it can serve as both a tumor promoting factor and a tumor suppressor, playing a double-edged sword role (20–22). However, the direct involvement of SMAD4 in regulating human NK cells against CRC cells and its impact on CRC progression remains unclear.

In this study, we illustrates that SMAD4 expression is downregulated in human NK cells within the CRC TME, and overexpression of SMAD4 enhance the cytotoxic ability of NK cells through upregulating the expression of the m^6^A reader protein YTHDF2 and promotes the expression of the NK activating receptor NKG2D. Consequently, targeting SMAD4 of NK cells may offer a promising therapeutic approach for CRC.

## 2 Materials and methods

### 2.1 The acquisition of pathological specimens from colorectal cancer patients

The para-cancerous and intra-cancerous samples of CRC patients were obtained from Xi’an International Medical Center Hospital, which with signed informed consent and approved by the Ethics Committee of Xi’an International Medical Center Hospital and Northwestern Polytechnical University for use of human samples.

### 2.2 Process for constructing the mouse colorectal cancer models

Eight-week-old female mice were utilized for the study, with mouse MC38 cells injected into the spleen at a concentration of 0.5×10^6^ cells per mouse. Liver tissue samples were collected and digested into single suspension cells at 0, 2, and 4 weeks post-injection. Flow cytometry was used to detect the number of NKp46^+^NK cells and to measure the expression of Smad4 in these NK cells. NKp46^+^NK cells were isolated using a flow cytometer, and qRT-PCR was employed to assess the mRNA expression of Smad4.

Eight-week-old female mice were treated with 5% DSS and AOM (15 mg/kg) to induce the progression of CRC (6). Colorectal adenocarcinoma tissue was collected at 4 and 8 weeks, digested into a single-cell suspension, and analyzed using flow cytometry to quantify the expression of Smad4 in NKp46^+^ NK cells. The animal experimentation was conducted in compliance with the guidelines set forth by Northwestern Polytechnical University Animal Care and Use Committee and adhered to an approved protocol.

### 2.3 Cell culture and infection

NK cells were maintained in NK cell specialized culture medium with recombinant IL-2 (25 ng/ml) and IL-15 (10ng/ml). HCT-116 cell was maintained in McCoy’s 5A basic medium (Invitrogen, USA) supplemented with 10% FBS. SW480 and SW620 cells were maintained in Roswell Park Memorial Institute 1640 (RPMI-1640) medium (Invitrogen, USA) with 10% FBS. The four kinds of cells were routinely tested for absence of mycoplasma using MycoAlertTM PLUS Mycoplasma Detection Kit (Lonza, MD, USA), incubated at 37°C in 5% CO_2_ and maintained with penicillin (100 U/mL) and streptomycin (100 µg/mL).

To overexpress human SMAD4 in NK cells, SMAD4 overexpression lentivirus (MOI of 30) or its control lentivirus (MOI of 30), which provided by Genechem (Shanghai, China), were used to infect human NK cells, separately. After 72 hours, NK cells from two groups were collected and co-cultured with human CRC cell lines (HCT-116, SW480, and SW620), separately. After 6 hours, total cells were collected and NK cells were sorted using a flow cytometer for subsequent testing.

In certain experimental conditions, human NK cells were infected with YTHDF2 interference lentivirus (MOI of 40), SMAD4 overexpression lentivirus plus YTHDF2 interference lentivirus. The mRNA and/or protein expression levels of METTL3, METTL14, FTO, ALKBH5, YTHDF1, YTHDF2, YTHDC1, NKG2D, PD-1, and TIGIT were assessed using qRT-PCR and flow cytometry.

### 2.4 Quantitative RT-PCR analysis

Quantitative RT-PCR (qRT-PCR) analysis was performed as described previously (12). Total RNA was isolated using the Trizol reagent (TaKaRa Biotechnology, China) according to the manufacturer’s protocol, cDNA synthesis and qRT-PCR were performed. The sequence of all primers are in **Supplementary Table 1**.

### 2.5 Cell surface and intracellular FACS analysis

Cell surface and intracellular fluorescence-activated cell sorting (FACS) analysis was performed as described previously (12). First, cells were incubated with antibodies targeting surface markers (CD56, NKG2D, NKp46), then washed and resuspended in Cytofix/Cytoperm solution (BD Biosciences) at 4°C for 20 min. Subsequently, the fixed and permeabilized cells were labeled with antibodies targeting intracellular markers (METTL3, METTL14, YTHDF2 or Smad4) for subsequent analysis using flow cytometry.

### 2.6 Cytotoxicity ability detection

In a 96-well plate, CRC cells (target cells) were co-cultured with NK cells (effector cells) at effector-to-target cell ratio (10:1) for 6 hours, and the cell surface and intracellular FACS analysis were used. First, cells were incubated with antibodies targeting surface markers (CD56, CD107a), then washed and resuspended in Cytofix/Cytoperm solution. Subsequently, the fixed and permeabilized cells were labeled with anti-human/mouse GZMB for subsequent analysis.

### 2.7 Statistical analysis

Statistical analysis was conducted using GraphPad Prism 7.0 software (USA), with comparisons between two independent or paired groups performed using the unpaired t-test. Results were reported as mean ± SD, with statistical significance defined as *P*<0.05.

## 3 Results

### 3.1 SMAD4 is lower expressed in CD56^+^NK cells of human CRC

**Figure 1:**
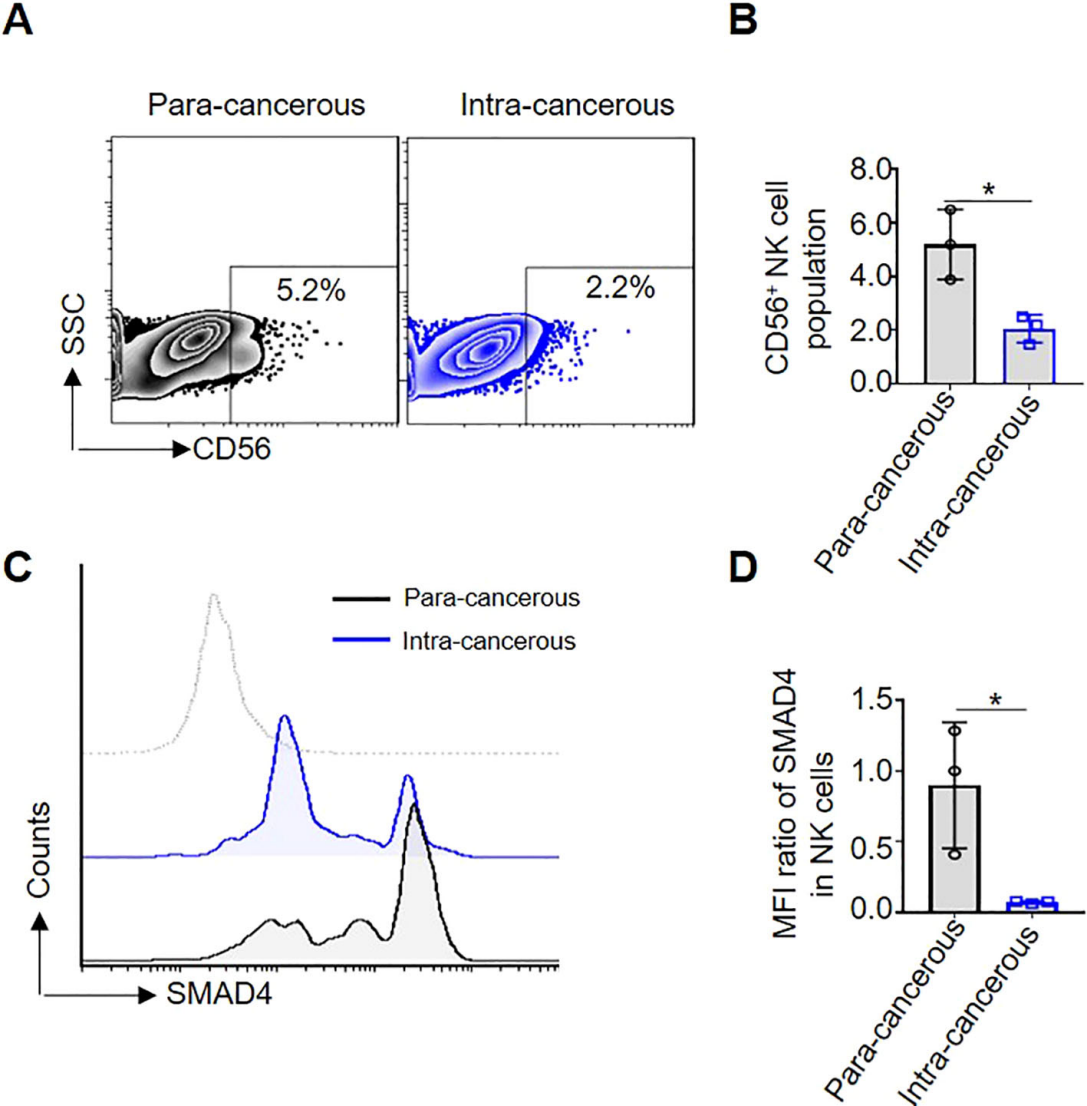
SMAD4 is lower expressed in CD56^+^NK cells of human CRC. **(A)** The proportion of CD56^+^NK cells in Para-cancerous and Intra-cancerous of CRC patient was detected through flow cytometry; **(B)** The statistics of NK cell population of three CRC patients; **(C)** SMAD4 protein expression of CD56^+^NK cells were detected in Para-cancerous and Intra-cancerous of CRC patient through flow cytometry; **(D)** Quantification of SMAD4 expression in NK cells of CRC patients: MFI ratio of SMAD4 in CD56^+^NK cells. N=3. Bars represent means ± SD, “*” *P <* 0.05.

To determine the role of SMAD4 in the CRC TME of human NK cells, we first compared the proportion of CD56^+^NK cells in para-cancerous and intra-cancerous of CRC patients. The results showed that the proportion of CD56^+^NK cells in intra-cancerous tissue is dramatically lower than that in para-cancerous tissue (**Figures 1A, B**). Secondly, compared with in para-cancerous of CRC patients, the protein expression of SMAD4 in CD56^+^NK cells of intra-cancerous showed a significantly lower level (**Figures 1C, D**). Taken together, we identified a global downregulation of SMAD4 in CD56^+^NK cells of intra-cancerous CRC patients.

### 3.2 Smad4 is expressed at lower levels in NK cells from mouse CRC tumor models

**Figure 2:**
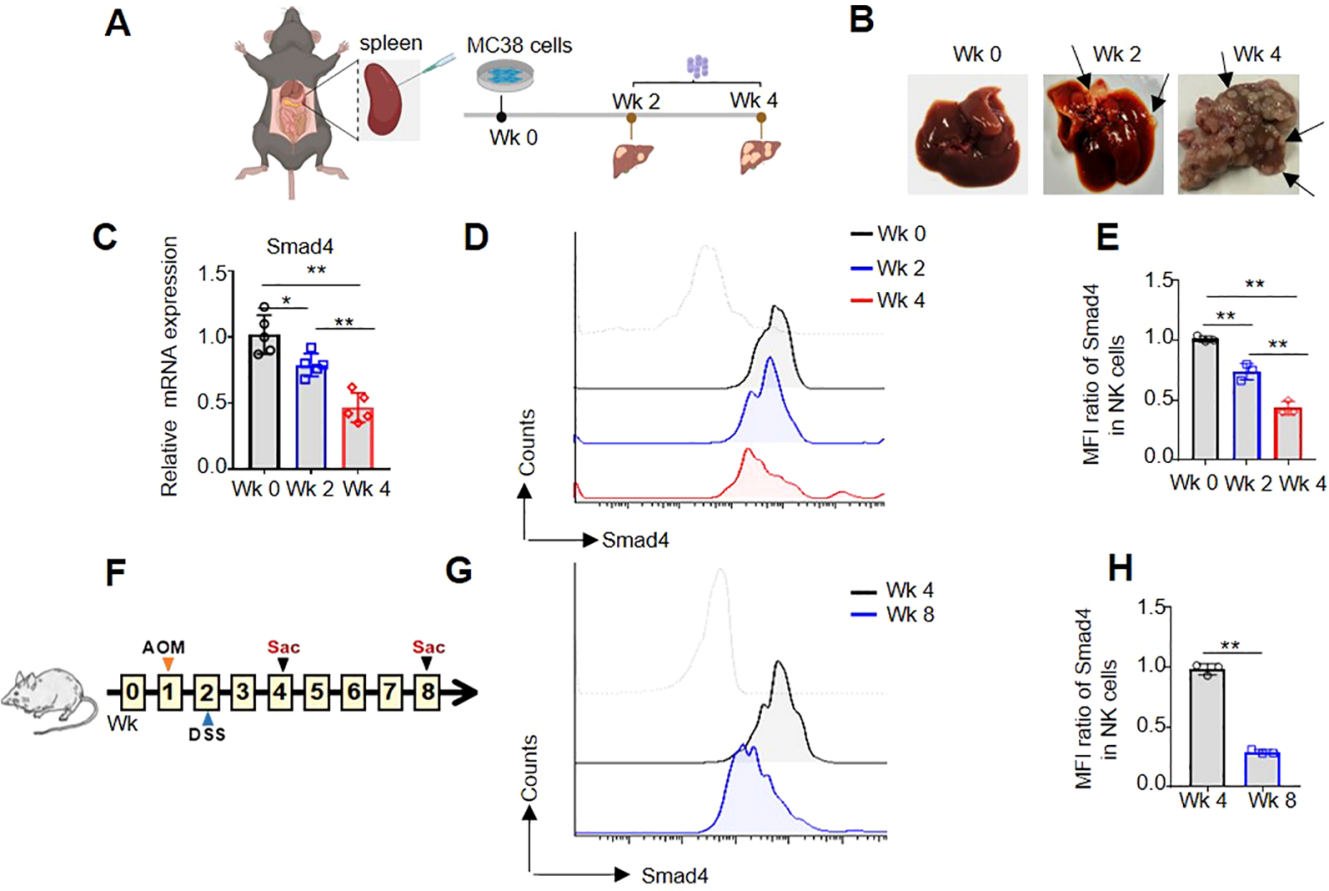
Smad4 is lower expressed in NK cells of mouse CRC tumor models. **(A)** Experimental timeline for MC38 tumor model; **(B)** CRC tumor nodes in liver; **(C)** The relative mRNA expression of Smad4 in NKp46^+^NK cells of liver; **(D)** Smad4 protein expressions in NKp46^+^NK cells were detected in liver of Wk 0, Wk 2 and Wk 4 through flow cytometry; **(E)** Quantification of **(D)**: MFI ratio of Smad4 in NKp46^+^NK cells; **(F)** Experimental timeline for AOM/DSS model; **(G)** Smad4 protein expressions in NKp46^+^NK cells were detected in colorectal tissue of Wk 4 and Wk 8 through flow cytometry; **(H)** Quantification of **(G)**: MFI ratio of Smad4 in NKp46^+^NK cells. N=3-5. Bars represent means ± SD, “**” *P <* 0.01, “*” *P <* 0.05.

In order to further investigate the expression of Smad4 in mouse colorectal cancer, two distinct mouse CRC models were developed. Firstly, a mouse model of MC38 colorectal cancer liver metastasis was constructed (**Figure 2A**), and the number of CRC tumor metastasis on the liver gradually increased, especially in the 4 weeks (Wk 4), indicating the successful construction of the MC38 liver metastasis model (**Figure 2B**). In liver metastatic CRC TME, flow cytometry and qRT-PCR analysis were used to detect the mRNA and protein levels of Smad4 in NKp46^+^NK cells. In comparison to liver NKp46^+^NK cells in 0 and 2 weeks (Wk 0, Wk 2), the mRNA and protein expression of Smad4 in liver NKp46^+^NK cells at Wk 4 exhibited a marked decrease (**Figures 2C-E**). Additionally, in the DSS/AOM CRC tumor model, flow cytometry was utilized to detect the protein expression of Smad4 in NKp46^+^NK cells. It was observed that the protein expression of Smad4 in NKp46^+^NK cells at 8 weeks (Wk 8) was significantly decreased compared to that at 4 weeks (Wk 4) (**Figures 2F-H**).When analyzed collectively, we observed a widespread decrease in Smad4 expression in mouse NK cells within CRC tumor TME.

### 3.3 SMAD4 is essential for the NK cell-mediated cytotoxicity against CRC cells

**Figure 3:**
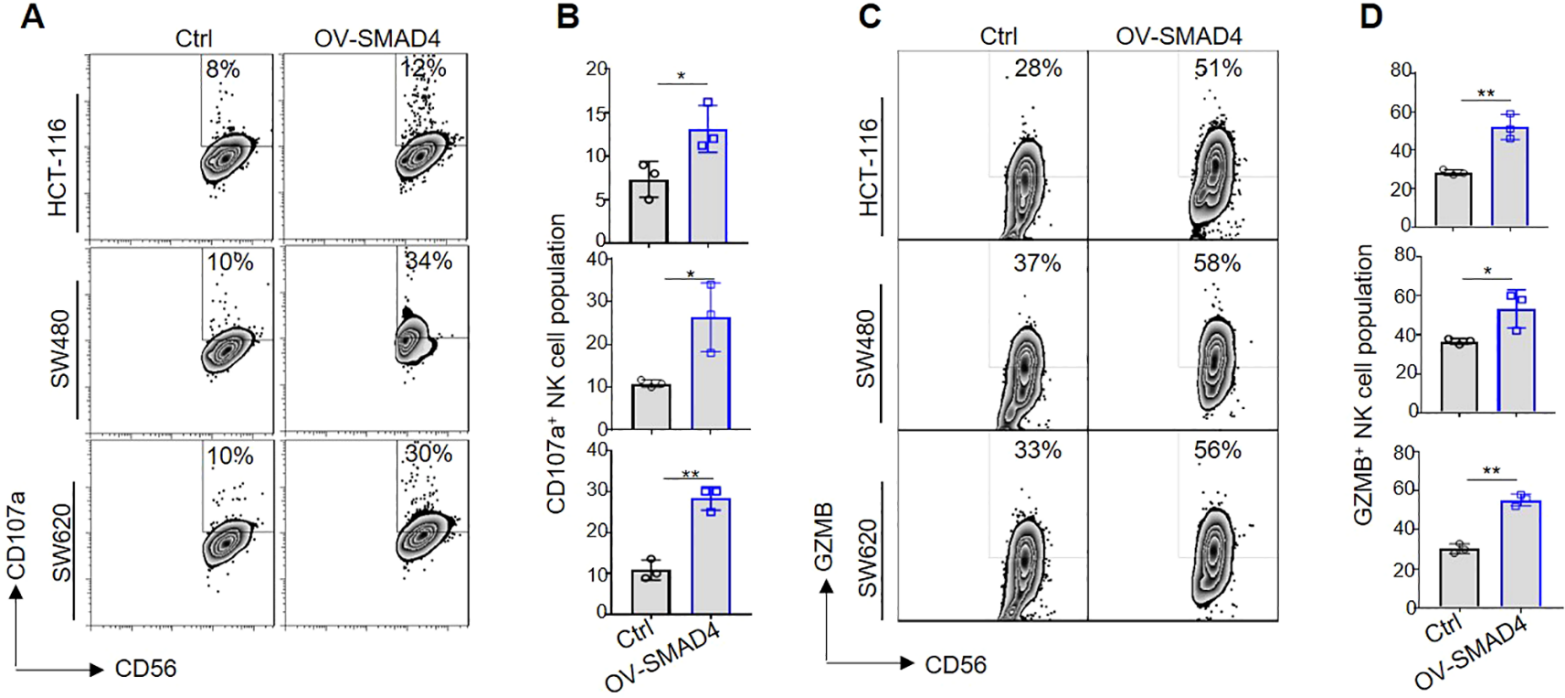
SMAD4 is essential for NK cell-mediated cytotoxicity against CRC cells. **(A-D)** The three CRC cell lines co-cultured with Ctrl NK cells or OV-SMAD4 NK cells, and the proportion of CD107a^+^ NK cells and GZMB^+^ NK cells were determined by flow cytometry. N=3. Bars represent means ± SD, “**” *P <* 0.01, “*” *P <* 0.05.

Cytotoxic activity is a crucial effector function of NK cells, which release significant quantities of lytic particles (e.g., GZMB) to induce lysis of tumor cells (12). Previous studies have demonstrated that specifically knockout of Smad4 in mouse NK cells accelerates the procession of melanoma (16). To investigate whether SMAD4 enhanced human NK cell-mediated cytotoxicity against CRC cells, the cytotoxicity assay were performed to assess the effects of SMAD4 overexpression on NK cell-mediated cytotoxicity towards various CRC cell lines, including HCT-116, SW480, and SW620. After co-culturing NK cells with three CRC cell lines, the expression of CD107a, a degranulation marker associated with tumor lysis capacity, were observed (12). When compared to the control group (Ctrl), the overexpression of SMAD4 group (OV-SMAD4) dramatically increased the expression of CD107a in human NK cells during co-cultured with HCT-116, SW480, and SW620 cell lines (**Figures 3A, B**). Furthermore, it was determined that the expression of GZMB, a crucial cytolytic effector, was also elevated in SMAD4-overexpressed NK cells compared to control NK cells (**Figures 3C, D**). In summary, these finding suggest that SMAD4 has the potential to enhance the cytotoxic activity of NK cells on CRC tumor cells.

### 3.4 Overexpression of SMAD4 promotes the m^6^A methyltransferases METTL3, METTL14 and m^6^A reading protein YTHDF2 expression in human NK cells

**Figure 4:**
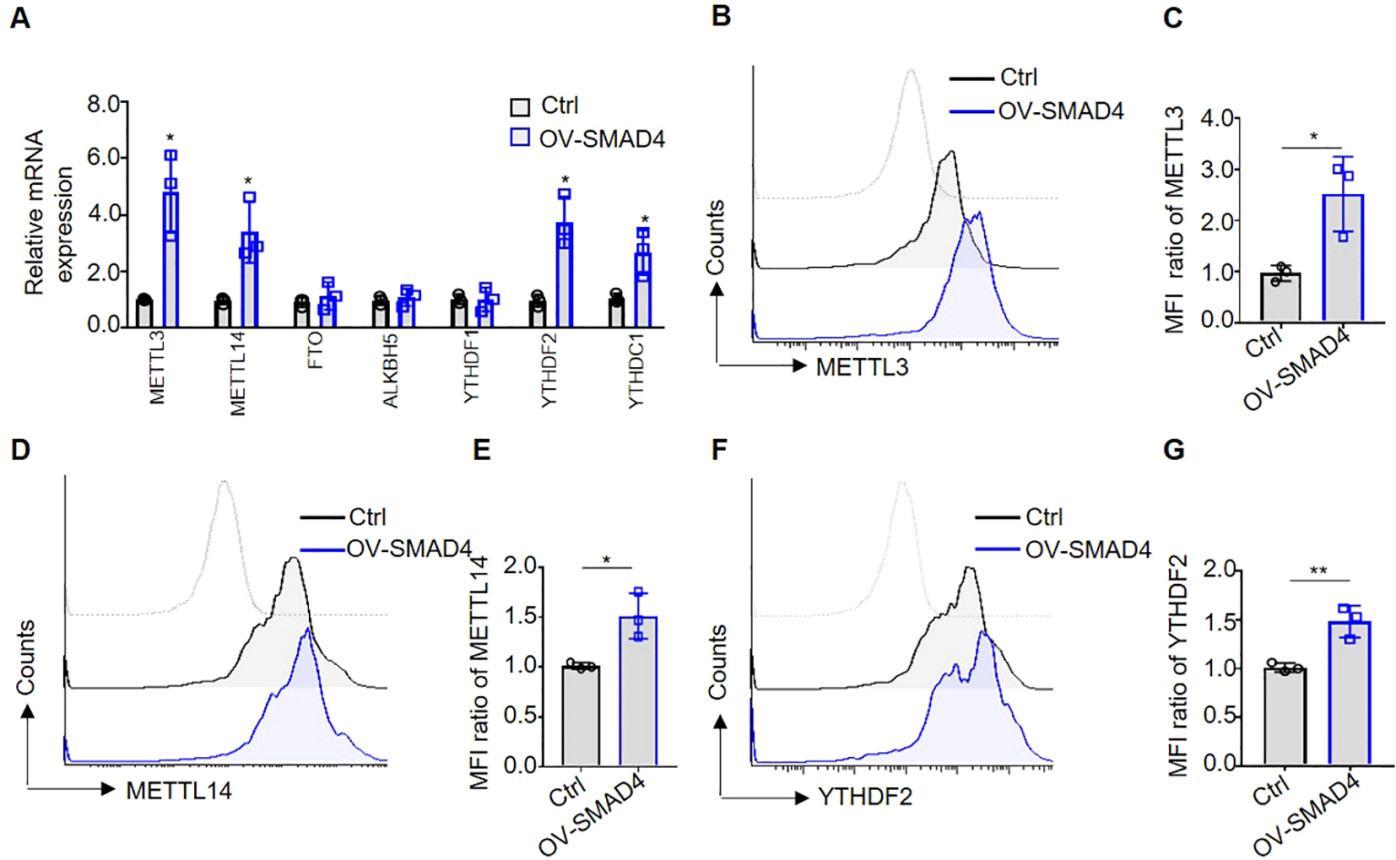
Overexpression of SMAD4 promotes the expression of m^6^A methyltransferases METTL3, METTL14 and m^6^A reading protein YTHDF2 in human NK cells. **(A)** The mRNA expressions of METTL3, METTL14, FTO, ALKBH5, YTHDF1, YTHDF2 and YTHDC1 in NK cells were detected by qRT-PCR; **(B-G)** The protein expressions of METTL3, METTL14 and YTHDF2 in NK cells were determined by flow cytometry. N=3. Bars represent means ± SD, “**” *P <* 0.01, “*” *P <* 0.05.

Recently researches have indicated that molecules involved in m^6^A methylation modification, such as METTL3 and YTHDF2, play an important role in promoting the anti-tumor activity of NK cells (23, 24). In order to further explore the molecular mechanisms underlying SMAD4-mediated NK cell cytotoxic against CRC cells, NK cells were transduced with either control lentivirus or SMAD4 overexpression (OV-SMAD4) lentivirus for 72 hours, followed by co-culture with CRC cells. Then, we detect the mRNA and/or protein expression levels of seven m^6^A methyltransferases regulators in NK cells. The findings indicate that the mRNA levels of m^6^A methyltransferases METTL3 and METTL14, as well as m^6^A reader proteins YTHDF2 and YTHDC1, were significantly upregulated in SMAD4 overexpression (OV-SMAD4) NK cells compared to control NK cells. Conversely, the mRNA levels of m^6^A demethylase FTO and ALKBH5, as well as m^6^A reader proteins YTHDF1, did not show significant changed (**Figure 4A**). Additionally, flow cytometry analysis was utilized to assess the protein expression of METTL3, METTL14, and YTHDF2 in NK cells. The findings indicate that the protein expression and mean fluorescence intensity (MFI) ratio of METTL3, METTL14, and YTHDF2 were significantly increase in OV-SMAD4 group compared to the control group (**Figures 4B-G**). In general, SMAD4 positively regulates the expression of METTL3 and METTL14, as well as YTHDF2 in human NK cells.

### 3.5 Overexpression of SMAD4 promote NKG2D expression in human NK cells

**Figure 5:**
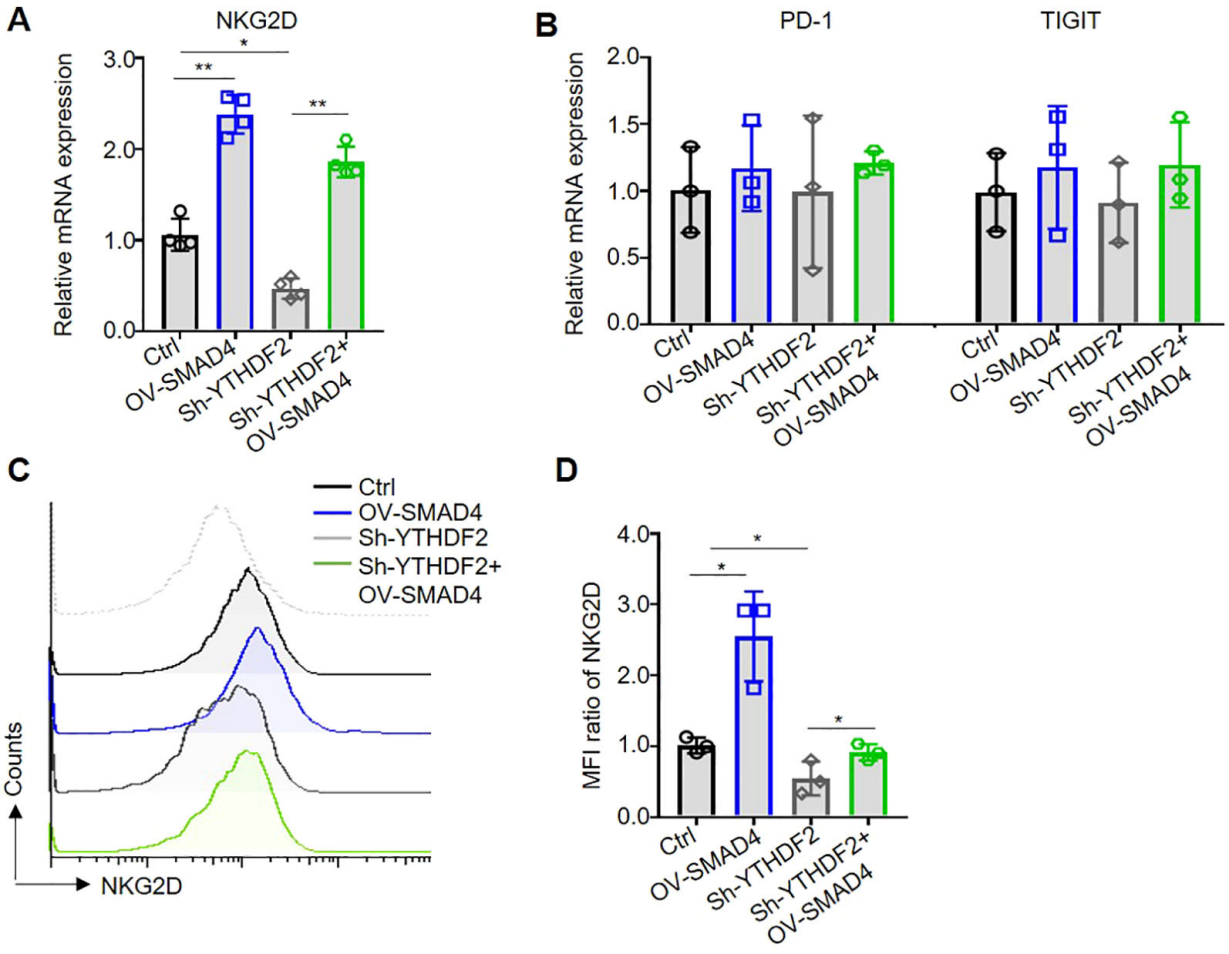
Overexpression of SMAD4 promotes NKG2D expression through YTHDF2 in human NK cells. **(A, B)** The mRNA expressions of NKG2D, PD-1 and TIGIT in NK cells were detected by qRT-PCR. **(C, D)** The protein expressions of NKG2D in NK cell surface were determined by flow cytometry. N=3-4. Bars represent means ± SD, “**” *P <* 0.01, “*” *P <* 0.05.

NK cell activating receptor NKG2D and immune checkpoints, such as PD-1 and TIGIT, are predominantly localized on the surface of NK cell membranes (24). In the TME of CRC, there is a notable upregulation of NK cell immune checkpoints or downregulation of activating receptors, which facilitates the evasion of tumor cells from NK cell-mediated cytotoxicity (5, 14). To explore the potential regulatory role of SMAD4 in the expression of NK cell inhibitory receptors and immune checkpoints, the mRNA levels of NKG2D, PD-1, and TIGIT were assessed in NK cells. Notably, overexpression of SMAD4 was found to promote NKG2D expression in human NK cells (**Figures 5A, B**). Subsequently, flow cytometry was utilized to detect the protein expression of NKG2D on the cell membrane surface. The finding indicate that the overexpression of SMAD4 remarkably promote the protein expression of NKG2D compared to the control (**Figures 5C, D**).

### 3.6 SMAD4 promotes NKG2D expression through YTHDF2

Subsequently, we further investigate the specific mechanism of SMAD4 mediated upregulation of NKG2D expression. Our finding reveals that interference with YTHDF2 (Sh-YTHDF2) led to a significant decrease in NKG2D mRNA expression compared to the control (Ctrl) group. Conversely, overexpression of SMAD4 plus interference with YTHDF2 (OV-SMAD4 + Sh-YTHDF2) resulted in rescued NKG2D mRNA expression, with no significant impact on the mRNA expression of PD-1 and TIGIT (**Figures 5A, B**). Furthermore, flow cytometry was employed to assess the protein expression of NKG2D. The findings indicate that interference with YTHDF2 notably decrease the protein expression of NKG2D in comparison to the control group. However, interference with YTHDF2 plus overexpression of SMAD4 resulted in the rescued NKG2D protein expression (**Figures 5C, D**). In conclusion, it can be inferred that SMAD4 can promotes NKG2D expression through YTHDF2.

### 3.7 SMAD4 enhances NK cell-mediated cytotoxicity on CRC cells through YTHDF2

**Figure 6:**
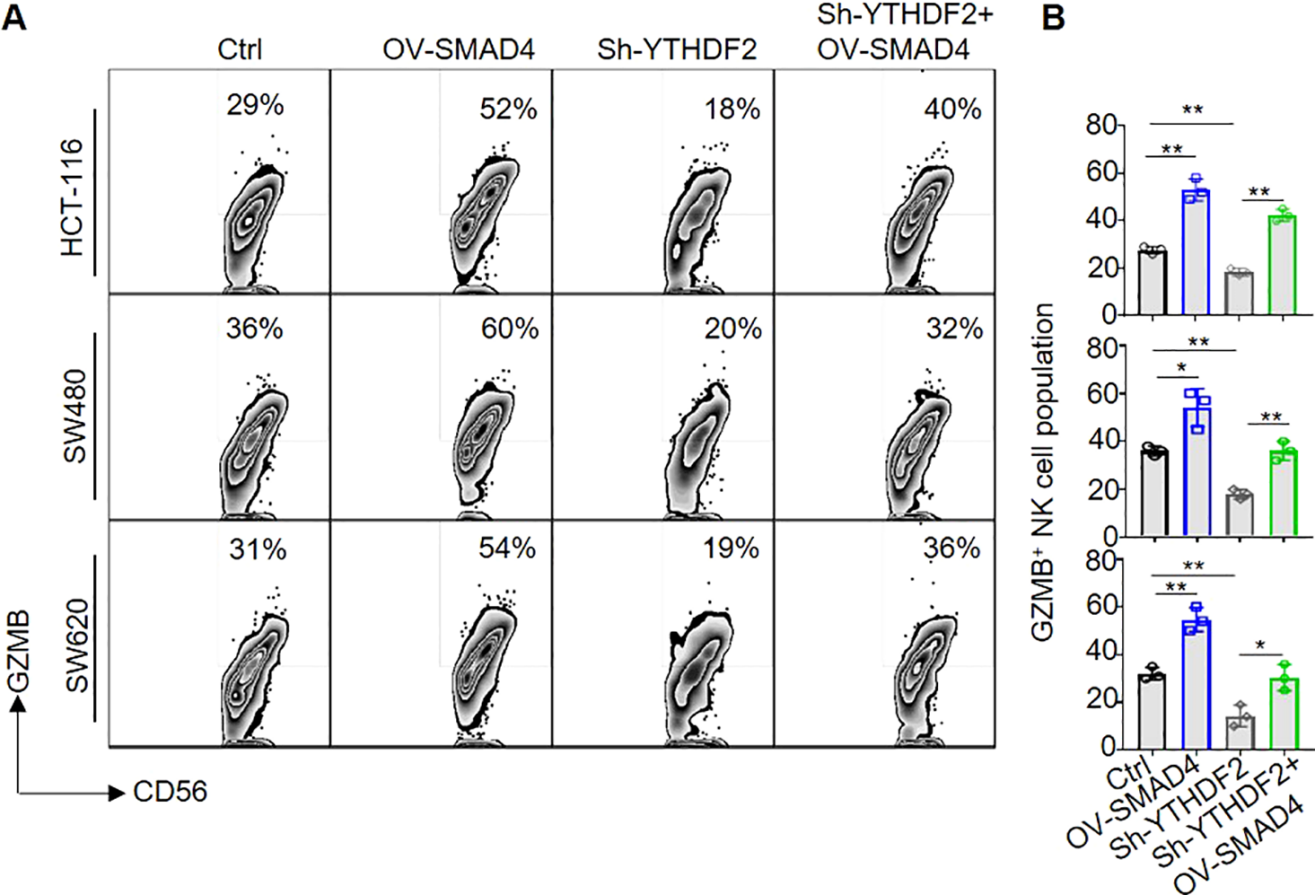
SMAD4 enhances NK cell-mediated cytotoxicity on CRC cells through YTHDF2. **(A, B)** The proportion of GZMB^+^ NK cells were determined by flow cytometry. N=3. Bars represent means ± SD, “**” *P <* 0.01, “*” *P <* 0.05.

Subsequently, in order to further investigate the potential role of SMAD4 in modulating the cytotoxicity of NK cells via YTHDF2, NK cells were transduced with lentiviral constructs encoding Ctrl, OV-SMAD4, Sh-YTHDF2, or OV-SMAD4 + Sh-YTHDF2 for 72 hours. Following this, the NK cells were co-cultured with CRC cells, and the expression of GZMB in NK cells was assessed using flow cytometry. The findings indicate that the protein expression of GZMB was notably decreased in the Sh-YTHDF2 group compared to the Ctrl group. However, compared with the Sh-YTHDF2 group, the OV-SMAD4+Sh-YTHDF2 group rescued the expression of GZMB. In conclusion, SMAD4 enhances NK cell-mediated cytotoxicity against CRC cells by modulating YTHDF2 (**Figures 6A, B**).

## 4 Discussion

**Figure 7:**
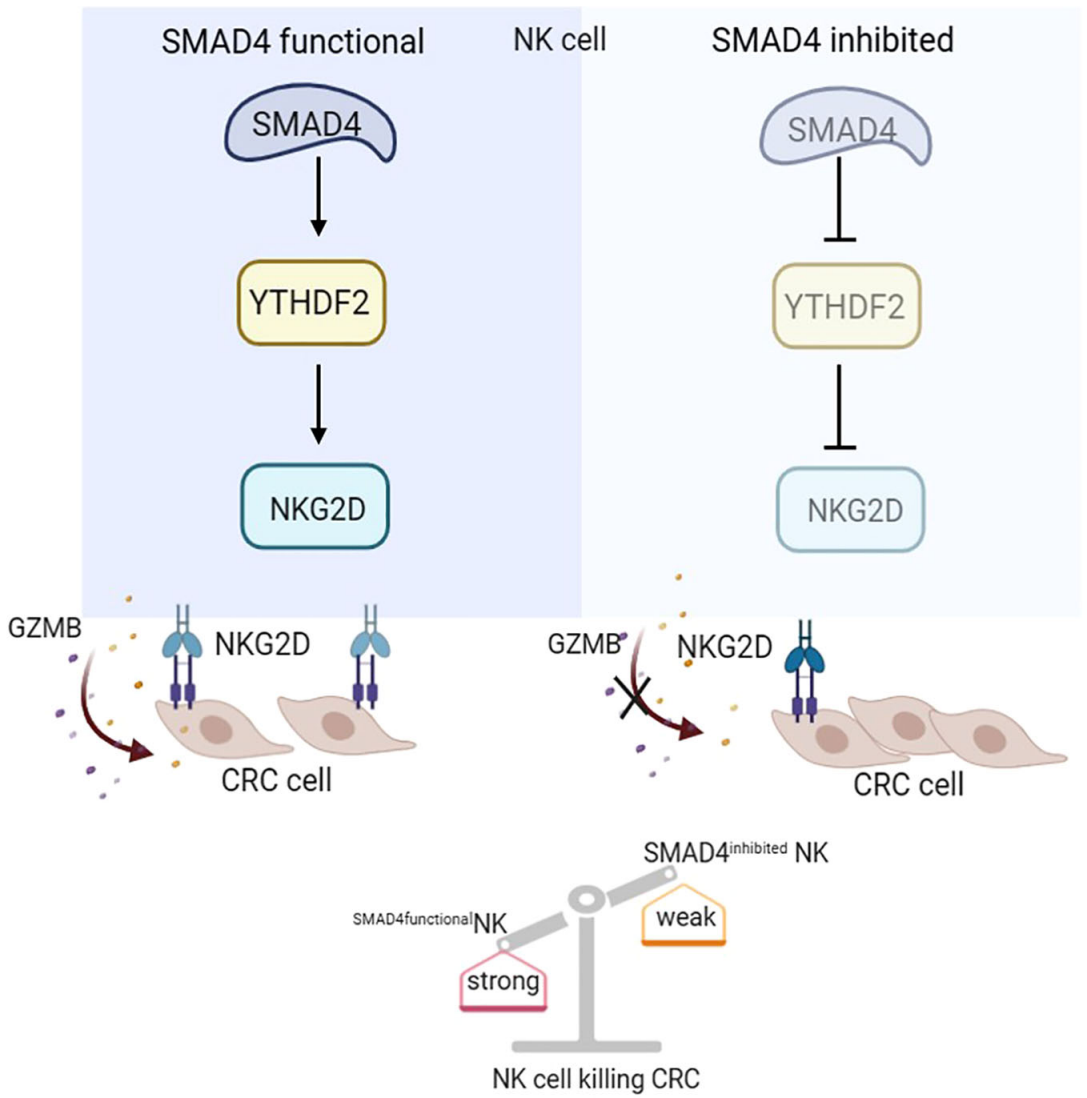
SMAD4 enhances the cytotoxicity of human NK cells against CRC cells through m^6^A reader YTHDF2.

In this study, we demonstrates that SMAD4 enhances the cytotoxic activity of human NK cells against CRC tumor cells. Our findings reveal that the m^6^A reader protein YTHDF2 is a downstream target of SMAD4 and it plays an important role in regulating NK cell cytotoxicity. Moreover, our research uncover that the upregulation of SMAD4 promotes the expression of the NK cell activating receptor NKG2D, thereby promoting the activation of the NKG2DL-NKG2D regulatory axis and enhancing the cytotoxic effect of NK cells to CRC cells. In summary, our study elucidates a novel mechanism by which SMAD4 modulates NK cells in their response to CRC cells, suggesting that SMAD4 could serve as a promising target for NK cell therapy on CRC (**Figure 7**).

Prior studies have demonstrated that aberrant expression or mutation of SMAD4 significantly influences the advancement of diverse tumors, including hematopoietic malignancies and solid tumors such as acute myeloid leukemia (25), pancreatic cancer (26), hepatocellular carcinoma (27), head and neck carcinoma (28) and CRC (29). In the context of CRC progression, the absence of SMAD4 hinders the infiltration of anti-tumor immune cells, thereby facilitating CRC recurrence and resistance to chemotherapy (30). Hu et al. recently employed lantern-shaped flexible nucleic acid origami technology to selectively deliver human SMAD4 mRNA into CRC cells, consequently impeding the growth of CRC (31). Additionally, two distinct research groups nearly concurrently discovered that mouse Smad4 suppresses viral infection and enhances the cytotoxicity of NK cells towards tumor cells, such as melanoma, with its regulatory mechanism being TGF-β independent, while the formation of a complex between Smad4 and JunB is synergistically enhance the transcription of GZMB (16, 19). Consistently, this study further elucidates the positive regulatory function of SMAD4 in facilitating the cytotoxicity of CRC cells by human NK cells. Our results indicate that SMAD4 likely plays a crucial role in activating NK cells to suppress CRC progression, and targeted activation of SMAD4 in human NK cells may represent a promising therapeutic approach for inhibiting CRC advancement.

NK cells, an essential component of the innate immune system, exhibit the ability to directly eliminate tumor cells through the secretion of cytotoxic molecules such as GZMB and PRF1. Recent studies have highlighted the significance of epigenetic modifications, specifically RNA modifications like m^6^A, in modulating the anti-tumor efficacy of NK cells. Song et al. demonstrated that the suppression of Mettl3 in NK cells results in diminished m^6^A levels and SHP-2 activity, consequently impairing the anti-tumor response of NK cells by reducing their sensitivity to IL-15 (23). Ma et al. demonstrated that knockout of Ythdf2 in mouse NK cells resulted in a notable decrease in GZMB expression, leading to diminished tumor-killing capabilities, particularly against melanoma. This suggests that Ythdf2 plays a crucial role in the anti-tumor function of NK cells (24). Our study further supports this notion that human SMAD4 enhances the cytotoxicity of NK cells, at least in part by upregulating YTHDF2 expression, thereby inhibiting the survival and proliferating of CRC cells. Subsequently, our research has provided functional evidence indicating that human SMAD4 positively regulates NKG2D expression through YTHDF2, leading to increased GZMB production when co-cultured with human CRC cells. Previous study in NK cells demonstrated that NKG2D can serve as a positively downstream target gene of YTHDF2, and specific knockout of Ythdf2 in mouse NK cells significantly inhibit the expression of NKG2D (24). CD8^+^ T cells have the similar cytotoxicity function of NK cells. Previous researcher showed that knockout of SMAD4 inhibit NKG2D mRNA expression (32). In summary, SMAD4-YTHDF2 regulatory axis regulates the expression of NKG2D in human NK cells.

In summary, our research elucidates that SMAD4 can enhance the cytotoxic activity of human NK cells against CRC tumor cells. Our findings offer new insights into the mechanisms by which SMAD4 promotes the cytotoxicity of NK cells against CRC tumors, specifically by upregulating YTHDF2 and NKG2D expression. These results underscore the potential therapeutic value of targeting the SMAD4 as a strategy for enhancing NK cell therapy on CRC.

## Data Availability

The original contributions presented in the study are included in the article/**Supplementary Material**. Further inquiries can be directed to the corresponding author.

## References

[1] Arnold M Sierra MS Laversanne M Soerjomataram I Jemal A Bray F. Global patterns and trends in colorectal cancer incidence and mortality. Gut. (2017) 66:683–91. doi: 10.1136/gutjnl-2015-310912 26818619

[2] Parikh AR Gonzalez-Gugel E Smolyakova N Jen MH Toms N Lin Y. Efficacy and safety of cetuximab dosing (biweekly vs weekly) in patients with KRAS wild-type metastatic colorectal cancer: a meta-analysis. Oncologist. (2022) 27:371–9. doi: 10.1093/oncolo/oyab030 PMC9074967 35522557

[3] Yaghoubi N Soltani A Ghazvini K Hassanian SM Hashemy SI. PD-1/PD-L1 blockade as a novel treatment for colorectal cancer. BioMed Pharmacother. (2019) 110:312–8. doi: 10.1016/j.biopha.2018.11.105 30522017

[4] Ben Hamouda S Essafi-Benkhadir K. Interplay between signaling pathways and tumor microenvironment components: A paradoxical role in colorectal cancer. Int J Mol Sci. (2023) 24:5600. doi: 10.3390/ijms24065600 36982677 PMC10057671

[5] Tallerico R Todaro M Di Franco S Maccalli C Garofalo C Sottile R. Human NK cells selective targeting of colon cancer–initiating cells: a role for natural cytotoxicity receptors and MHC class I molecules. J Immunol. (2013) 190:2381–90. doi: 10.4049/jimmunol.1201542 23345327

[6] Pan P Kang S Wang Y Liu K Oshima K Huang YW. Black raspberries enhance natural killer cell infiltration into the colon and suppress the progression of colorectal cancer. Front Immunol. (2017) 8:997. doi: 10.3389/fimmu.2017.00997 28861089 PMC5561013

[7] Lanuza PM Vigueras A Olivan S Prats AC Costas S Llamazares G. Activated human primary NK cells efficiently kill colorectal cancer cells in 3D spheroid cultures irrespectively of the level of PD-L1 expression. Oncoimmunology. (2018) 7:e1395123. doi: 10.1080/2162402X.2017.1395123 29632716 PMC5889279

[8] Xiao L Cen D Gan H Sun Y Huang N Xiong H. Adoptive transfer of NKG2D CAR mRNA-engineered natural killer cells in colorectal cancer patients. Mol Ther. (2019) 27:1114–25. doi: 10.1016/j.ymthe.2019.03.011 PMC6554529 30962163

[9] Xu C Liu D Chen Z Zhuo F Sun H Hu J. Umbilical cord blood–derived natural killer cells combined with bevacizumab for colorectal cancer treatment. Hum Gene Ther. (2019) 30:459–70. doi: 10.1089/hum.2018.011 29914273

[10] Marcus A Mao AJ Lensink-Vasan M Wang L Vance RE Raulet DH. Tumor-derived cGAMP triggers a STING-mediated interferon response in non-tumor cells to activate the NK cell response. Immunity. (2018) 49:754–763. e754. doi: 10.1016/j.immuni.2018.09.016 30332631 PMC6488306

[11] Crinier A Narni-Mancinelli E Ugolini S Vivier E. SnapShot: natural killer cells. Cell. (2020) 180:1280–1280. e1281. doi: 10.1016/j.cell.2020.02.029 32200803

[12] Li X Dong W Nalin AP Wang Y Pan P Xu B. The natural product chitosan enhances the anti-tumor activity of natural killer cells by activating dendritic cells. Oncoimmunology. (2018) 7:e1431085. doi: 10.1080/2162402X.2018.1431085 29872557 PMC5980345

[13] Glasner A Levi A Enk J Isaacson B Viukov S Orlanski S. NKp46 receptor-mediated interferon-γ production by natural killer cells increases fibronectin 1 to alter tumor architecture and control metastasis. Immunity. (2018) 48:107–119. e104. doi: 10.1016/j.immuni.2017.12.007 29329948

[14] Zhang Z Su T He L Wang H Ji G Liu X. Identification and functional analysis of ligands for natural killer cell activating receptors in colon carcinoma. Tohoku J Exp Med. (2012) 226:59–68. doi: 10.6120/tjem.226.59 22189020

[15] Aashaq S Batool A Mir SA Beigh MA Andrabi KI Shah ZA. TGF-β Signaling: A recap of SMAD-independent and SMAD-dependent pathways. J Cell Physiol. (2022) 237:59–85. doi: 10.1002/jcp.30529 34286853

[16] Wang Y Chu J Yi P Dong W Saultz J Wang Y. SMAD4 promotes TGF-β–independent NK cell homeostasis and maturation and antitumor immunity. J Clin Invest. (2019) 128:5123–36. doi: 10.1172/JCI121227 PMC6205382 30183689

[17] Huss DJ Winger RC Cox GM Guerau-de-Arellano M Yang Y Racke MK. TGF-β signaling via Smad4 drives IL-10 production in effector Th1 cells and reduces T-cell trafficking in EAE. Eur J Immunol. (2011) 41:2987–96. doi: 10.1002/eji.201141666 PMC3478765 21728174

[18] Hu Y Lee YT Kaech SM Garvy B Cauley LS. Smad4 promotes differentiation of effector and circulating memory CD8 T cells but is dispensable for tissue-resident memory CD8 T cells. J Immunol. (2015) 194:2407–14. doi: 10.4049/jimmunol.1402369 PMC4337487 25637015

[19] Cortez VS Ulland TK Cervantes-Barragan L Bando JK Robinette ML Wang Q. SMAD4 impedes the conversion of NK cells into ILC1-like cells by curtailing non-canonical TGF-β signaling. Nat Immunol. (2017) 18:995–1003. doi: 10.1038/ni.3809 28759002 PMC5712491

[20] David CJ Huang YH Chen M Su J Zou Y Bardeesy N. TGF-β tumor suppression through a lethal EMT. Cell. (2016) 164:1015–30. doi: 10.1016/j.cell.2016.01.009 PMC4801341 26898331

[21] Bardeesy N Cheng Kh Berger JH Chu GC Pahler J Olson P. Smad4 is dispensable for normal pancreas development yet critical in progression and tumor biology of pancreas cancer. Genes Dev. (2006) 20:3130–46. doi: 10.1101/gad.1478706 PMC1635148 17114584

[22] Malkoski SP Wang XJ. Two sides of the story? Smad4 loss in pancreatic cancer versus head-and-neck cancer. FEBS Lett. (2012) 586:1984–92. doi: 10.1016/j.febslet.2012.01.054 PMC3285395 22321641

[23] Song H Song J Cheng M Zheng M Wang T Tian S. METTL3-mediated m6A RNA methylation promotes the anti-tumour immunity of natural killer cells. Nat Commun. (2021) 12:5522. doi: 10.1038/s41467-021-25803-0 34535671 PMC8448775

[24] Ma S Yan J Barr T Zhang J Chen Z Wang LS. The RNA m6A reader YTHDF2 controls NK cell antitumor and antiviral immunity. J Exp Med. (2021) 218:e20210279. doi: 10.1084/jem.20210279 34160549 PMC8225680

[25] Quéré R Karlsson G Hertwig F Rissler M Lindqvist B Fioretos T. Smad4 binds Hoxa9 in the cytoplasm and protects primitive hematopoietic cells against nuclear activation by Hoxa9 and leukemia transformation. Blood. (2011) 117:5918–30. doi: 10.1182/blood-2010-08-301879 21471525

[26] Xia X Wu W Huang C Cen G Jiang T Cao J. SMAD4 and its role in pancreatic cancer. Tumor Biol. (2015) 36:111–9. doi: 10.1007/s13277-014-2883-z 25464861

[27] Hernanda PY Chen K Das AM Sideras K Wang W Li J. SMAD4 exerts a tumor-promoting role in hepatocellular carcinoma. Oncogene. (2015) 34:5055–68. doi: 10.1038/onc.2014.425 25531314

[28] Lin LH Chang KW Cheng HW Liu CJ. SMAD4 somatic mutations in head and neck carcinoma are associated with tumor progression. Front Oncol. (2019) 9:1379. doi: 10.3389/fonc.2019.01379 31867281 PMC6909744

[29] Yan P Klingbiel D Saridaki Z Ceppa P Curto M McKee TA. Reduced expression of SMAD4 is associated with poor survival in colon cancer. Clin Cancer Res. (2016) 22:3037–47. doi: 10.1158/1078-0432.CCR-15-0939 26861460

[30] Wasserman I Lee LH Ogino S Marco MR Wu C Chen X. SMAD4 loss in colorectal cancer patients correlates with recurrence, loss of immune infiltrate, and chemoresistance. Clin Cancer Res. (2019) 25:1948–56. doi: 10.1158/1078-0432.CCR-18-1726 PMC6421131 30587545

[31] Hu M Feng C Yuan Q Liu C Ge B Sun F. Lantern-shaped flexible RNA origami for Smad4 mRNA delivery and growth suppression of colorectal cancer. Nat Commun. (2023) 14:1307. doi: 10.1038/s41467-023-37020-y 36894556 PMC9998469

[32] Igalouzene R Hernandez-Vargas H Benech N Guyennon A Bauché D BarraChina C. SMAD4 TGF-β–independent function preconditions naive CD8^+^ T cells to prevent severe chronic intestinal inflammation. J Clin Invest. (2022) 132:e151020. doi: 10.1172/JCI151020 35426367 PMC9012287

