# Supplementary material for: SMAD4 enhances the cytotoxic efficacy of human NK cells against colorectal cancer cells via the m6A reader YTHDF2
Source: Front Immunol. 2024 Oct 7;15:1440308. doi: 10.3389/fimmu.2024.1440308 (PMC11494605; doi:10.3389/fimmu.2024.1440308)
Supplement: Supplementary file 1 [file Table1.docx]

**Supplementary Tabel 1**

**PCR Primers Used in the Study**

| **Name/Target gene** | **Primer sequence** |
| --- | --- |
| Mouse Smad4 | F: 5’- CAGCCATAGTGAAGGACTGTTGC-3’;  R: 5’- CCTACTTCCAGTCCAGGTGGTA -3’ |
| Human METTL3 | F: 5’- CTATCTCCTggCACTCgCAAgA -3’;  R: 5’- gCTTgAACCgTgCAACCACATC -3’ |
| Human METTL14 | F: 5’- CTgAAAgTgCCgACAgCATTgg -3’;  R: 5’- CTCTCCTTCATCCAgATACTTACg -3’ |
| Human ALKBH5 | F: 5’- CCAgCTATgCTTCAgATCgCCT -3’;  R: 5’- ggTTCTCTTCCTTgTCCATCTCC -3’ |
| Human FTO | F: 5’- CCAgAACCTgAggAgAgAATgg-3’;  R: 5’- CgATgTCTgTgAggTCAAACgg -3’ |
| Human YTHDF1 | F: 5’- CAAgCACACAACCTCCATCTTCg -3’;  R: 5’- gTAAgAAACTggTTCgCCCTCAT -3’ |
| Human YTHDF2 | F: 5’- TAgCCAgCTACAAgCACACCAC -3’;  R: 5’- CAACCgTTgCTgCAgTCTgTgT -3’ |
| Human YTHDC1 | F: 5’- TCAggAgTTCgCCgAgATgTgT -3’;  R: 5’- AggATggTgTggAggTTgTTCC -3’ |
| Human NKG2D | F: 5’- GGTATGAGAGCCAGGCTTCTTG-3’;  R: 5’- GAATGGAGCCATCTTCCCACTG-3’ |
| Human PD-1 | F: 5’- AAGGCGCAGATCAAAGAGAGCC -3’;  R: 5’- CAACCACCAGGGTTTGGAACTG -3’ |
| Human TIGIT | F: 5’- TGGTGGTCATCTGCACAGCAGT -3’;  R: 5’- TTTCTCCTGAGGTCACCTTCCAC -3’ |
